# The association between the site of back pain and number of painful sites with daily activities, seeking healthcare, and medication use among school adolescents

**DOI:** 10.3389/fpain.2025.1459232

**Published:** 2025-01-30

**Authors:** Tatiana Rehder Gonçalves, Mauro Felippe Felix Mediano, Rosely Sichieri, Diana Barbosa Cunha

**Affiliations:** ^1^ Department of Epidemiology, Institute of Social Medicine, State University of Rio de Janeiro, Rio de Janeiro, Brazil; ^2^ Institute of Studies in Public Health, Federal University of Rio de Janeiro (UFRJ), Rio de Janeiro, Brazil; ^3^ Evandro Chagas National Institute of Infectious Disease, Oswaldo Cruz Foundation, Rio de Janeiro, Brazil

**Keywords:** back pain, neck pain, thoracic pain, low back pain, adolescent, daily activities

## Abstract

**Introduction:**

The potential consequences of back pain (BP) are poorly described in adolescents. This study evaluated the association between BP sites (neck, thoracic and low back) and number of painful sites with missed school classes, interference in physical activities, seeking healthcare, and medication use among school adolescents.

**Methods:**

This cross-sectional study included 350 students (5th to 9th grade) of a public school in Brazil. Information on BP site and outcomes were self-reported. Logistic regression analyses were performed.

**Results:**

Participants reporting at least one painful site in spine were 74.9% (*n* = 262), with mean age of 12.73 ± 1.67 (55.7% were girls). Most of them reported pain in two sites (*n* = 100; 28.6%) and the most frequent pain site was neck (*n* = 223; 63.7%). Thoracic and low BP were associated with missing school classes, interference in physical activities, seeking healthcare, and medication use, while neck pain showed no association. The number of painful sites was associated with daily activities and healthcare with those gradients increasing with the number of painful sites.

**Conclusion:**

Thoracic and low BP were associated with daily activities, seeking healthcare, and medication use in early adolescence.

## 1 Introduction

Back pain (BP), encompassing any discomfort in the neck, thoracic and low back, is an important public health problem that accounts to a high prevalence and functional impact among adults, being one of the most expensive conditions for the public health system in many countries around the world (1, 2). BP prevalence has been increasing not only in adult population but also among children and adolescents, reaching up to 40% during their lifetime (3).

In adults, the impact of BP has been reported by some studies that demonstrate work absenteeism, functional impairments, high utilization of healthcare system, limitation to perform functional activities, participation restrictions and long-term incapacity (1, 2, 4). However, despite the growing number of studies assessing prevalence and factors associated with BP during adolescence in recent years, few have focused on the potential consequences of BP on different aspects of daily activities and healthcare utilization in adolescents - an area of importance given the potential link between BP during this phase and later life (5, 6). The reasons for this gap in the literature might be explained by the misperception that BP during adolescence is only a minor health condition with no major consequences on daily activities (7), even though other studies have demonstrated that pain is associated with increased rates of healthcare system use (8, 9).

Moreover, most studies that investigated the potential consequences of BP in adolescents have focused on low BP (10), neglecting other possible back painful sites in spine which are also prevalent and may also have consequences on daily activities (11–13). Although the presence of multiple BP sites may exacerbate the negative consequence of BP on daily activities and lead to greater healthcare utilization and medication use in adolescents, potentially due to greater pain sensitization, studies exploring the relationship between the number of painful BP sites and its consequences in this population remains limited in literature (14). In this setting, studies that provide evidence to guide public actions in health and education, with the aim of minimizing the impacts of back pain, promoting the well-being of adolescents and preventing future complications, ensuring a healthy transition to adulthood are necessary. Therefore, the aim of the present study was to evaluate the association between BP sites and the number of painful sites in the spine with daily activities (missed school classes and interference in physical activities), seeking healthcare, and medication use among school adolescents.

## 2 Methods

Adolescents from 5th to 9th grades enrolled in a public school in the city of Niteroi (Rio de Janeiro, Brazil) were invited to participate. Those with age other than 10–18 years, with physical or mental impairments that prevented from filling in the questionnaire or performing anthropometric measurements, and pregnant were excluded. Sample size was calculated based on a BP prevalence of 30% (13), with an estimated error of 5% and 95% confidence interval and a further increase of 30% due to a nonresponse rate, being necessary 323 adolescents.

Before the inclusion of the adolescents in the study, parents/guardian signed an informed consent. The study was approved by the Research Ethics Committee of the Social Medicine Institute of the State University of Rio de Janeiro (CAAE 10471313.2.0000.5260).

### 2.1 Exposure - pain assessment

**Figure 1:**
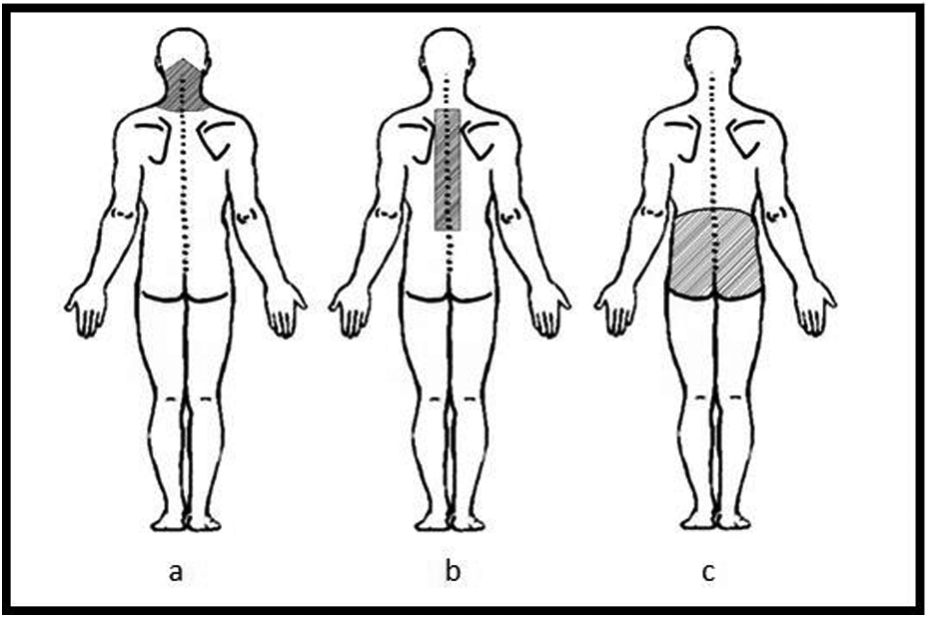
Body map including the shaded spinal area (a. Neck; b. Thoracic; c. Low Back) shown beside each pain question.

Information about lifetime BP was obtained based on the following question: “How many times have you had pain in …?” (1) neck, (2) thoracic and (3) low back pain. Response options were dichotomized into “Yes” (for “Often”, “Once in a while” and “Once or twice”) and “No” (for “Never”). A body map including the shaded spinal area was shown beside each question (Figure 1) (11–13, 15). The number of painful sites in the spine was obtained by summing up the presence of pain on neck (NP), thoracic (TP), and low back pain (LBP) regions.

### 2.2 Outcomes

Information on daily activities (missed school classes and interference in physical activities), seeking health care and medication use were self-reported using a structured questionnaire, as follows: “Have you stayed home from school because of neck or (middle or low) back pain?”, “Has neck or (middle or low) back pain sometimes stopped you from doing physical activity (sport or recreation)?”, “Have you been to a doctor, physiotherapist or hospital because of neck or (middle or low) back pain?”, “Have you ever taken medicine due to neck or (middle or low) back pain?”. The response options were dichotomized into “Yes” (for “Often”, “Once in a while” and “Once or twice”) and “No” (for “Never”) (15, 16).

### 2.3 Data analysis

Descriptive statistics for categorical variables comprised absolute and relative frequencies. Since all questions about daily activities and health care were tied to participants that reported back pain (e.g, missed school due to pain), the regression analysis included only those adolescents that reported pain in any of the spine's sites (*n* = 262). Binary logistic regression models were fitted to evaluate the associations between neck, thoracic and low back pain with daily activities (missed school classes and interference in physical activities), seeking healthcare, and medication use adjusted for potential confounders (sex, age, and race). Each outcome was included as a dependent variable in a separate logistic regression model that included painful sites (neck, thoracic and low back) and number of painful sites in spine as independent variables, separately. Tests for linear trends were performed by modelling number of painful sites in spine as a continuous variable. All analyses were performed using Stata 13.0.

## 3 Results

The study included 350 adolescents with mean age of 12.7 (±1.6) years, 51.4% (*n* = 180) were girls, 50.6% (*n* = 177) self-reported their race as mulatto, 19.1% (*n* = 67) as white, 14.6% (*n* = 51) as black, and 15.7% (*n* = 55) others. Characteristics of BP sites, number of painful sites in the spine and pain impact are described in Table 1. The most cited site of pain was neck (*n* = 223; 63.7%) and most of the adolescents reported pain in two sites of the spine (*n* = 100; 28.6%). Of those adolescents that reported pain (74.9%, *n* = 262; mean age = 12.7 ± 1.7; 55.7% girls, *n* = 146), 29.0% (*n* = 76) reported missing school classes, 35.9% (*n* = 94) interference in physical activities, 22.5% (*n* = 59) seeking for health care, and 26.3% (*n* = 69) medication usage.

**Table 1:** Site of pain, number of painful sites and outcomes (missed school, interference in physical activities, seeking health care and medication usage) among adolescents.

	Total (*n* = 350)
Site of pain	*n* (%)
Neck	223 (63.7)
Thoracic	190 (54.3)
Low back	121 (34.6)
Number of painful sites	*n* (%)
No pain	88 (25.1)
One	76 (21.7)
Two	100 (28.6)
Three	86 (24.6)
Outcomes (*n* = 262)	*n* (%)
Missed school	76 (29.0)
Interference in physical activities	94 (35.9)
Seeking health care	59 (22.5)
Medication usage	69 (26.3)

The associations between NP, TP and LBP with daily activities, seeking healthcare, and medication use are described in Table 2. The presence of TP and LBP were associated with higher odds of missing school classes (OR 2.61; 95% CI 1.25–5.42 and OR 2.37; 95% CI 1.34–4.20), interference in physical activities (OR 2.62; 95% IC 1.35–5.11 and OR 2.19; 95% CI 1.28–3.75), seeking healthcare (OR 2.72; 95% IC 1.20–6.19 and OR 1.94; 95% CI 1.06–6.19), and medication use (OR 3.81; 95% CI 1.60–9.01 and OR 3.24; 95% CI 1.76–5.96), respectively. No significant associations were observed between NP and daily activities and health care.

**Table 2:** Frequency and Odds Ratios (OR) of the association between sites of pain in the spine with missed school, interference in physical activities, seeking health care and medication usage among adolescents.

	Total (*n* = 262)		
	*n* (%)	Chi-squared *p*-value	OR (95% CI)^a^
Missed school			
Neck pain			
No	7 (18.0)	0.099	Reference
Yes	69 (30.9)		2.28 (0.94 to 5.54)
Thoracic pain			
No	11 (15.3)	**0**.**003**	Reference
Yes	65 (34.2)		**2.61 (1.25 to 5.42)**
Low back pain			
No	28 (19.9)	**<0**.**001**	
Yes	48 (39.7)		**2.37 (1.34 to 4.20)**
Interference in physical activities			
Neck pain			
No	11 (28.2)	0.279	Reference
Yes	83 (37.2)		1.72 (0.79 to 3.74)
Thoracic pain			
No	15 (20.8)	**0**.**002**	Reference
Yes	79 (41.6)		**2.62 (1.35 to 5.11)**
Low back pain			
No	37 (26.2)	**<0**.**001**	Reference
Yes	57 (47.1)		**2.19 (1.28 to 3.75)**
Seeking health care			
Neck pain			
No	10 (25.6)	0.613	Reference
Yes	49 (22.0)		0.90 (0.40 to 2.01)
Thoracic pain			
No	8 (11.1)	**0**.**007**	Reference
Yes	51 (26.8)		**2.72 (1.20 to 6.19)**
Low back pain			
No	23 (16.3)	**0**.**009**	Reference
Yes	36 (29.8)		**1.94 (1.06 to 6.19)**
Medication usage			
Neck pain			
No	62 (27.8)	0.197	Reference
Yes	7 (18.0)		1.88 (0.76 to 4.64)
Thoracic pain			
No	7 (9.72)	**<0**.**001**	Reference
Yes	62 (32.6)		**3.81 (1.60 to 9.01)**
Low back pain			
No	21 (14.9)	**<0**.**001**	Reference
Yes	48 (39.7)		**3.24 (1.76 to 5.96)**

Values in bold are those statistically significant.

^a^ Logistic regression model adjusted by sex, race, age and pain in the other spine sites.

The greater number of painful sites (two or three painful sites) in the spine was negatively associated with daily activities, seeking healthcare, and medication use in a dose-response manner (*p*-value for trend < 0.01 for all analyses) (Table 3).

**Table 3:** Frequency and Odds Ratios (OR) of the association between number of painful sites in the spine with missed school, interference in physical activities, seeking health care and medication usage among adolescents.

	Total (*n* = 262)			
	*n* (%)	Chi-squared *p*-value	OR (95% CI)^a^	*p*-value for trend
Missed school				
One site	7 (9.2)	**<0**.**001**	Reference	**<0**.**001**
Two sites	32 (32.0)		**4.87 (1.97 to 11.99)**	
Three sites	37 (43.0)		**7.72 (3.15 to 18.96)**	
Interference in physical activities				
One site	10 (13.2)	**<0**.**001**	Reference	**<0**.**001**
Two sites	43 (43.0)		**5.22 (2.36 to 11.57)**	
Three sites	41 (47.7)		**6.40 (2.86 to 14.30)**	
Seeking health care				
One site	9 (11.8)	**0**.**012**	Reference	**0**.**003**
Two site	23 (23.0)		**2.35 (1.00 to 5.53)**	
Three sites	27 (31.4)		**3.60 (1.55 to 8.40)**	
Medication usage				
One site	4 (5.3)	**<0**.**001**	Reference	**<0**.**001**
Two sites	27 (27.0)		**6.33 (2.08 to 19.27)**	
Three sites	38 (44.2)		**13.68 (4.56 to 41.05)**	

Values in bold are those statistically significant.

^a^ Logistic regression model adjusted by sex, race and age.

## 4 Discussion

The present study demonstrated an important association of TP and LBP with daily activities, seeking healthcare, and medication use among school adolescents, while NP showed no association. The greater number of painful sites was associated with an increased strength of association in a dose- response manner.

Most studies investigating the impact of BP among adolescents focus on LBP. Jones et al. studied the influence of recurrent LBP in adolescents and observed an important association with missing school, visiting professional care and stopping sports or physical activity (17). These results are in accordance with ours regarding the negative association of LBP with daily activities and similar to those found by Sullivan et al., which revealed that 20% of adolescents who reported LBP demonstrated important impacts including medication use, care seeking and school absenteeism (18). Also, Masiero et al. observed that 40% of adolescents attending high school sought medical advice because of their LBP and almost 30% reported disabling LBP (19). Furthermore, a longitudinal study demonstrated a growing impact of LBP during the transition from adolescence to young adulthood (20). In this setting, some authors suggested that LBP is a potential disabling condition even during young ages (17, 18, 21).

Studies have shown that thoracic pain (TP) is a common condition that should not be underestimated in children and adolescents (22, 23). However, to the best of our knowledge, the significant associations with daily activities, seeking healthcare, and medication use identified in our study have not been previously demonstrated. Since thoracic spine contributes in a variety of body movements, TP can cause a great discomfort to perform many activities of daily living, leading to a greater impairment (24). On the other hand, although NP demonstrated a high prevalence, no significant association was observed. These results contrast with those observed in the Global Burden of Disease 2017 study, in which NP was one of the most important contributors of disability in adolescents as measured by years lived with disability (25). These conflicting results can be attributed to different measurements of pain impact, with the present study focusing on specific questions about missing school, participation in physical activity (e.g sports), seeking healthcare, and medication use, activities that may be less directly impacted by NP. Additionally, adolescents with NP may be able to manage their symptoms in ways that do not significantly disrupt these specific activities, possibly leading to a lower perceived impact compared to pain in other locations of spine (26).

A study examining the impact of pain in any place of the body on daily activities among children and adolescents found that pain accounts for 80% of the reported restrictions in daily activities (27). The number of painful sites has also been associated with physical and social activities in adults, but there is only few evidence among adolescents (28). Holden et al. examined the patterns of pain in adolescents and its association with sports participation and health-related quality of life. They found that multi-site pain (with a high proportion of BP) was associated with a low health-related quality of life (29). Oliveira et al. observed that 35% of school adolescents reporting neck or low BP sought health care in Brazil with those experiencing daily activity limitations being more likely to do so (30). In the same way, our study found an important association between the number of painful sites with daily activities, seeking healthcare, and medication use, indicating that a higher number of painful sites is linked to greater severity of these conditions. In our understanding, more critical than identifying the specific combinations of painful sites was recognizing the presence of multiple painful sites in the body, which characterizes diffuse pain and suggests the possibility of central sensitization.

The present study has some limitations. The lack of information about the duration of pain precludes to disentangle the influence of chronic, subacute, or acute BP episodes; however, for lifetime experiences, the characterization of the duration is difficult to measure. Furthermore, questions about pain impact are not related to a specific episode of pain. We acknowledge that the lack of data on pain intensity, as well as psychosocial and environmental factors, limits our ability to fully explore the complex relationship between BP and its potential consequences in adolescents. Future studies should include these parameters to provide a more comprehensive understanding of this issue. Although cross-sectional study design precludes temporal associations, in our study, the outcome questions are tied to participants that reported BP, reducing potential reverse-causality bias. Strengths of the present study included the evaluation of all spine sites (neck, thoracic and low back) and the number of painful sites at the same time, a condition that commonly happens in clinical practice.

To conclude, the increasing prevalence of BP in children and adolescents is a growing public health concern due to its functional limitations, social costs, and potential progression into adulthood. This study shows that TB and LBP had an important association with daily activities, seeking healthcare, and medication use even during early adolescence, indicating that strategies to prevent and manage BP at this age are already necessary. These findings underscore the need for targeted interventions aiming to prevent and manage BP among Brazilian adolescents, with the potential to mitigate its long-term physical, psychological, and socioeconomic consequences, fostering better health outcomes and reducing the burden on healthcare system. Longitudinal studies including a more detailed evaluation of BP and its associated functional impacts are still necessary.

## Data Availability

The de-identified raw data supporting the conclusions of this article will be made available by the authors upon reasonable request.
